# Priorities for Decreasing Morbidity and Mortality in Children With Advanced HIV Disease

**DOI:** 10.1093/cid/ciy013

**Published:** 2018-03-04

**Authors:** Lisa Frigati, Moherdran Archary, Helena Rabie, Martina Penazzato, Nathan Ford

**Affiliations:** 1 Department of Paediatrics and Child Health, Tygerberg Hospital and Stellenbosch University, Cape Town; 2 University of KwaZulu Natal, Nelson R Mandela School of Medicine, Berea, South Africa; 3 HIV Department, World Health Organization, Geneva, Switzerland

**Keywords:** children, advanced HIV disease, HIV

## Abstract

Early mortality and morbidity remain high in children initiating antiretroviral therapy (ART), especially in sub-Saharan Africa. Many children still present with advanced human immunodeficiency virus (HIV) disease. Tuberculosis, pneumonia, and severe bacterial infections are the main causes of hospital admission in HIV-infected children. In contrast to adults with advanced HIV disease, cryptococcal disease is not common in childhood, although there is a peak in infancy and adolescence. Interventions such as TB screening in symptomatic children, and isoniazid and cotrimoxazole prophylaxis should be implemented. There is evidence suggesting that rapid initiation (within 1 week) of ART in children with severe malnutrition or those with advanced HIV disease admitted to hospital is not beneficial and should be delayed until their condition has been stabilized. Research informing the prevention of severe bacterial infections, the management of pediatric immune reconstitution inflammatory syndrome, and other potential strategies to decrease morbidity and mortality in HIV-infected children are urgently needed.

Mortality in human immunodeficiency virus (HIV)–infected children with advanced disease initiating antiretroviral therapy (ART) is high, particularly in sub-Saharan Africa [1]. Despite significant progress in scaling up access to ART globally and a shift toward initiating ART in all children, an important proportion of HIV-infected children continue to present to services with advanced HIV disease, and there is high mortality within the first 6 months of initiating ART in Africa and Asia [2–4]. There is also some evidence that high mortality among children with advanced HIV disease is not limited to low-resource settings: a recent study examining trends in mortality and AIDS-defining events after ART initiation among children and adolescents with perinatal HIV in Europe and Thailand found that, of 94 deaths, 43 (46%) occurred within the first 6 months and 79 of the 174 (45%) first AIDS-defining events occurred within 6 months after ART initiation [5].

The major causes of mortality and morbidity in children with advanced HIV disease are opportunistic infections (notably tuberculosis [TB] and pneumocystis pneumonia [PCP]), bacterial infections, diarrheal disease, and severe acute malnutrition (as well as malaria in endemic areas) [6, 7]. While early diagnosis and treatment remain priorities, new strategies are needed to reduce this burden of early mortality.

Increasingly, as with adults, children are re-presenting to health facilities with advanced HIV after a period of having interrupted their ART or while taking an ART regimen that is no longer effective due to development of drug resistance. Strategies to decrease mortality need to take this growing population of children into account.

The World Health Organization (WHO) recently released guidelines focusing on adults and children with advanced HIV disease with the aim of reducing mortality in this population [8]. These guidelines focus on providing an enhanced package of prophylactic, diagnostic, and therapeutic interventions for those initiating ART with advanced disease, as well as providing recommendations on rapid initiation of ART. The adult data informing these guidelines are mostly from 2 trials that evaluated packages of interventions to decrease the mortality in patients presenting with advanced disease [9, 10]. There is limited evidence specifically related to children, and no randomized controlled trial in those <5 years of age.

This article highlights the differences between the guideline recommendations for adults and children with regard to the definition of advanced disease in children <5 years, the differences in prevalent opportunistic infections and how this relates to a preventive package of care, when to initiate ART in severely ill children, vaccination of children with advanced HIV, and other interventions that may reduce morbidity in children.

## DEFINING ADVANCED DISEASE IN CHILDREN

Severe immunological suppression in children <5 years of age is classified based on age-related CD4 categories [11]. However, young children have an increased risk of disease progression and mortality regardless of their clinical or immune staging. Data from the International Epidemiology Databases to Evaluate AIDS (IeDEA) pediatric cohort showed that 80% of children who started ART either had advanced clinical and/or immunological staging [12]. In the Children With HIV Early Antiretroviral Therapy (CHER) study, 24% (129/532) of children between 6 and 12 weeks of age who were screened could not be enrolled because of significant clinical or laboratory findings or a CD4 percentage <25% [13]. In another study of children presenting to hospital with severe acute malnutrition, 25% were WHO immunological stage 1 or 2. (M. Archary, personal communication). This finding emphasizes the fact that higher CD4 percentages in children can misrepresent the severity of clinical illness. Of note, 25% of children starting ART at <5 years of age in the IeDEA cohort had no known WHO stage or CD4 cell count or percentage, highlighting the difficulty in immunological staging in children <5 years of age in resource-limited settings (G. Patten for the IeDEA cohort, personal communication).

These and other data led the WHO Guideline Development Group to recommend that, for the purposes of the 2017 guideline, all children should be classified as having advanced disease.

### CAUSES OF MORBIDITY AND MORTALITY IN HIV-INFECTED CHILDREN

The main causes of morbidity and mortality in children on ART are AIDS-related conditions (TB and PCP) and bacterial infections followed by malnutrition and wasting, hematological disturbances, and in Africa, malaria.

Mortality among hospitalized HIV-infected children averages 14% [7], with some studies reporting that more than one-quarter of HIV-infected hospitalized children had died [14, 15]. One study among adolescents in Zimbabwe found that in-hospital mortality was >3 times higher among HIV-infected (23%) compared with HIV-uninfected patients (7%), with death significantly associated with low CD4 cell count [16]. In Cape Town, South Africa, 13% of HIV-infected infants <18 months of age admitted to a tertiary facility died [17]. Mortality from pneumonia was higher in younger children with HIV than in older children and adults [6].

A recent systematic review and meta-analysis estimated that TB incidence in pediatric HIV cohorts ranged from 0.3 to 25.3 per 100 person-years [18]. This review suggested that while ART decreases the risk of TB infection in children, its full protective potential is only evident 1–2 years after starting ART. TB is especially common in the first 3 months after starting ART [19], and it is estimated that up to 15% of children starting ART in sub-Saharan African countries will develop TB [20]. Routine isoniazid prophylaxis has been shown to decrease morbidity and mortality in HIV-infected children [21], although it is currently not recommended for children <1 year of age without a known TB contact [22]. The importance of providing access to prevention in children with TB cannot be overemphasized. Screening for TB in children with HIV remains problematic as respiratory symptoms are common. Using culture as the reference standard, studies have shown that the sensitivity of the Xpert MTB/RIF assay for pulmonary TB was 2–3 times higher than that of smear microscopy when testing induced sputum, nasopharyngeal aspirates, or gastric aspirate lavages [23–25]. Urine lipoarabinomannan is recommended for use in children with advanced HIV disease [26], but has been shown to have lower diagnostic accuracy in HIV-infected children regardless of CD4 cell count [27]. Where chest radiography is available, this should be included in the package available to clinicians to screen for pediatric TB in symptomatic HIV-infected children.

Studies done in Zambia and South Africa in the pre-ART era showed a high prevalence of PCP [28, 29], with infants <6 months of age being especially vulnerable regardless of CD4 cell count. Despite improved access to ART, PCP remains a common cause of hospital admission due to undiagnosed HIV in infants. Cotrimoxazole has been shown to prevent PCP and a range of other bacterial infections as well as preventing malaria in endemic regions [30]. In African children on ART randomized to continue or stop cotrimoxazole after a median of 2.1 years of ART, continued therapy did not prevent death but did reduce hospitalization due to malaria and other infections [31]. It also prevented hospital admissions in the pre-ART era [30, 32]. Of note, in studies of severe pneumonia in HIV-infected young infants, cytomegalovirus (CMV) infection may play an important role in mortality [33, 34]. Initiation of ART during pregnancy may increase early transmission to children, causing congenital infection but also infection at a very young age with potential serious disease [35]. Diagnostic challenges and the high cost of therapy for CMV limit the ability to effectively reduce death in these children.

Although there is a peak of diagnosis in infancy and preadolescence [36], in contrast to adults, cryptococcal disease is rare especially in children <5 years of age. A retrospective review of HIV-infected children with cryptococcosis at a tertiary hospital in Cape Town, South Africa, over 7 years identified only 7 children, among whom the median age of cryptococcal diagnosis was 9.3 years (ranging from 6 to 13.6 years) [37]. In a laboratory-based survey performed in South Africa, cryptococcal disease incidence was estimated at 47 per 100000 children [38], and within 2 pediatric trial cohorts no cases of cryptococcal disease were reported in children <5 years of age [39]. In adults, reflex screening for cryptococcal antigen in those with a CD4 count <100 cells/μL has been implemented in South Africa, but there are no data to inform at what age to start screening in older children and adolescents.

High rates of bacteremia have been described in children starting ART, especially within the first 3 months of treatment [40, 41]. A recent study in 82 children with malnutrition admitted to a tertiary hospital in Durban, South Africa, showed that 6% of admission blood cultures were positive. Healthcare-associated infections were predominately gram-negative (39/43), and 39.5% were extended-spectrum β-lactamase positive. No pathogen was specifically associated with mortality [42].

Immune reconstitution inflammatory syndrome (IRIS) has been described in children and may be the underlying mechanism of morbidity presenting within the first 6 months after starting ART [43]. Tuberculosis is a common cause of mortality in the first 3 months after starting ART [44]. Recent data from the P1073 study in sub-Saharan Africa and India that enrolled 202 children <72 months of age showed that 18.6% of the cohort had an episode of IRIS, with some children experiencing >1 episode. Bacille Calmette-Guérin IRIS, TB disease, and oral candidiasis were the most common causes and 3.5% episodes of IRIS were defined as complicated and resulted from TB or CMV (M. Cotton, personal communication). The prevention and management of IRIS has been studied in adults. South African studies assessing the use of prednisone in the management and prevention of TB-IRIS found that clinicians should check susceptibility of tuberculosis in IRIS cases and that initiating steroids prior to ART in severely immunosuppressed patients may prevent serious IRIS [45]. However, no data exist for children.

In the REALITY trial, 4% (72) of participants were between 5 and 17 years of age and received the same package of prophylactic interventions as the adult group. No child had cryptococcal disease, 1 child had oral *Candida* at enrollment, and no new cases were detected during the trial. One death was reported due to probable bacterial pneumonia [9]

## WHEN TO START ART IN CHILDREN ADMITTED TO HOSPITAL

Three recent studies have suggested that early initiation of ART may not be the first priority in sick children. In the first study, a randomized trial from South Africa, young children (median age 23 months) with severe acute malnutrition were randomized to receive ART within 14 days of admission or, for ART to be delayed until nutritional recovery (and after 2 weeks; median time, 23 days). The results suggested that a reasonable delay in ART improved immune recovery, led to faster viral suppression, and improved anthropometric measures [46]. In the second study, a randomized trial from Kenya, HIV-infected hospitalized children (median age 23 months) were randomized to ART start within 48 hours vs 7–14 days. While there was no difference in mortality between treatment arms, the authors concluded that rapid treatment was safe and prompt initiation of ART is essential to reduce the very high mortality observed overall, with 21% of children dying during 6 months of follow-up [1]. The third study from Malawi enrolled children with uncomplicated malnutrition and suggested that earlier ART initiation (within 21 days) improved nutritional recovery [47], although this was not a randomized controlled trial. Overall, while ART initiation is a priority, particularly for children aged <5 years, children who present with malnutrition or other illnesses and those requiring hospitalization need to be stabilized first.

Overall, data on the use of a package of interventions in children >5 years of age are limited and no data are available in children <5 years old.

Based on previous recommendations included in the WHO 2016 consolidated ART guidelines, the package of screening and prophylaxis interventions for children consists of screening and diagnosis of TB, and preventive measures such as cotrimoxazole prophylaxis and isoniazid prophylaxis. However, cryptococcal antigen screening and fluconazole prophylaxis are not recommended due to limited data and the lower disease burden, reducing the likely cost-effectiveness of these approaches.

Increased pill or syrup burden is a particular concern for children and, where possible, fixed-dose combination formulations should be utilized, including the new fixed-dose combination of cotrimoxazole, isoniazid, and pyridoxine [48], which is a scored tablet and has shown good bioavailability in children. A half-dose scored tablet is still needed for children <5 years of age. Whether the current package (Table 1) is sufficiently adapted to the specific leading causing of mortality in children remains an important question for future research, particularly to address the high rates of bacteremia in the first 3 months of treatment. Approaches that may decrease early death and morbidity include presumptive treatment of or additional prophylaxis for bacterial infections, availability of ganciclovir, early treatment of hematological disorders, prompt treatment of oral/esophageal *Candida*, oral rehydration for diarrhea, and more frequent home visits/follow-up or growth monitoring.

**Table 1.** Components of the Package of Care for Human Immunodeficiency Virus (HIV)–Infected Adolescents and Children With Advanced HIV Disease

Intervention	Component	Adolescents	Children
Diagnosis	Sputum Xpert MTB/RIF assay as the first test for symptomatic children/adolescents	Yes	Yes
	Urine LF-LAM assay for symptomatic children/adolescents	Yes	Yes^a^
	Cryptococcal antigen screening	Yes (CD4 <100 cells/μL)	No
Prophylaxis and preemptive treatment	Cotrimoxazole prophylaxis	Yes	Yes
	TB preventive therapy	Yes	Yes^b^
	Fluconazole preemptive therapy for cryptococcal antigen–positive people without evidence of meningitis	Yes (CD4 <100 cells/μL)	Not applicable as screening not recommended
ART initiation	Rapid initiation after initial stabilization (unless evidence of tuberculous meningitis or cryptococcal meningitis^c^)	Yes	Yes^c^

Abbreviations: ART, antiretroviral therapy; LF-LAM, lateral flow lipoarabinomannan; TB, tuberculosis.

^a^There are limited data for children.

^b^For children <12 months, only those with a history of TB contact should receive TB preventive treatment if the evaluation shows no TB disease.

^c^Consideration should be given to delaying ART in children with severe acute malnutrition.

Finally, routine interventions recommended by WHO for the general pediatric population such as deworming, malaria prophylaxis, and vitamin A supplementation should all be provided. Childhood vaccinations may also prevent bacteremia, diarrhea, and pneumonia and should be reinforced.

Overall, it remains critical to ensure that interventions to minimize early mortality are implemented in countries with a high burden of pediatric HIV.

## Notes

***Financial support.*** This supplement was supported by funds from the Bill & Melinda Gates Foundation.

***Supplement sponsorship.*** This article appears as part of the supplement "Advanced HIV Disease," sponsored by the World Health Organization..

***Potential conflicts of interest.*** All authors: No reported conflicts of interest. All authors have submitted the ICMJE Form for Disclosure of Potential Conflicts of Interest. Conflicts that the editors consider relevant to the content of the manuscript have been disclosed.
